# Knowledge, Attitude, and Perception of Parents in the Najran Region Toward the Use of Herbal Medicine in the Treatment of Asthma in Their Children

**DOI:** 10.7759/cureus.51308

**Published:** 2023-12-29

**Authors:** Mohammed J Alzahrani, Abeer M AlSaadi, Ali Taher T Alyami, Abdulmajeed Saad S Alshahrani, Mohammed H Alshaiban, Raed Saeed J Alwadai, Mohammed H Alqurayshah, Saleh Hamad S Alkulayb, Nasser S Al Hyder, Saleh Alshaiban

**Affiliations:** 1 Department of Pediatrics, College of Medicine, Najran University, Najran, SAU; 2 Department of Pediatrics, King Salman Bin Abdulaziz Medical City, Medina, SAU; 3 College of Medicine, Najran University, Najran, SAU

**Keywords:** najran city, asthma, alternative medicine, herbal medicine, herbs

## Abstract

Background: Asthma is a prevalent and persistent condition affecting the respiratory system, defined by the presence of fluctuating and reversible symptoms associated with the restriction of airflow, heightened sensitivity of the bronchial tubes, and inflammation within the airways. Saudi Arabia has a high prevalence of this chronic illness, making it one of the most often seen conditions in the country. A popular therapeutic approach within the realm of complementary and alternative medicine for individuals with asthma is the use of herbal medications. Nevertheless, the efficacy of these medicines in treating asthma is often supported by inadequate data.

Objectives: This study aimed to determine the knowledge, attitude, and perception of parents toward the use of herbal medicines in the treatment of asthma in their children in the Najran Region.

Methods: In this cross-sectional study, the participants received an electronic self-administered survey via social media channels. The survey instrument comprises a set of questions that have been gathered from prior studies that have comparable research aims. The questionnaire sheet will contain three parts. Data were analyzed with IBM SPSS Statistics for Windows, Version 22 (Released 2013; IBM Corp., Armonk, New York, United States). The sample and outcome variables were summarized using the descriptive statistics of frequencies, percentages, means, and standard deviations. Data were analyzed by type of provider as appropriate, and the Pearson chi-square test was used to compare the observed data; the paired sample t-test was used to test the difference between the means of the pretest and post-test.

Results: Fifteen percent of the respondents personally suffer from asthma, while 85.0% do not. Additionally, 25.7% of the respondents indicated that one of their children suffers from asthma, while 74.3% stated that their children do not suffer from asthma. The data also show that the statement "Asthma symptoms are dry cough, shortness of breath, and wheezing" received a 51.4% "Strongly Agree" response, 37.9% "Agree," 9.3% "Neutral," 0.9% "Disagree," and 0.5% "Strongly Disagree." The majority of respondents (51.4%) reported obtaining information about asthma from doctors. A notable portion of respondents (35.5%) reported using herbs or folk medicines to treat asthma in their children. A substantial portion of respondents (44.9%) reported not using medicinal herbs at all. Despite this, 41.6% of respondents expressed belief in the idea of taking herbs as a treatment.

Conclusion: In conclusion, the knowledge, attitude, and perception of parents toward the use of herbal medicine in the treatment of asthma in their children are multifaceted and influenced by cultural, social, and individual factors. Understanding these dynamics is essential for developing culturally sensitive healthcare interventions and policies that align with the beliefs and practices of the community.

## Introduction

Asthma is a common chronic disorder of the airways, characterized by variable reversible and recurring symptoms related to airflow obstruction, bronchial hyper-responsiveness, and underlying inflammation. It is one of the most common chronic diseases in Saudi Arabia, affecting more than two million Saudis. Its impact is manifested in patients, their families, and the community as a whole in terms of lost work and school days, poor quality of life, frequent emergency department visits, hospitalizations, and deaths [1]. Studies that used the ISAAC determined prevalence rates of 4%-33.7% for asthma diagnosed by physicians among children in Saudi Arabia [2]. In a more recent study carried out by Professor Alqahtani, through questionnaire-based assessments, the prevalence rates for childhood asthma were determined where it was found that the overall prevalence of physician-diagnosed asthma was 27.5% among Saudi school children in Najran City [3].

Conventional treatments for allergic asthma include steroids, leukotriene antagonists, bronchodilators, and the most recent anti-IgE antibody. All these drugs still with certain shortcomings such as side effects, effectiveness, and cost. It has become more and more important to develop novel therapeutic approaches for the treatment of allergic asthma [4]. Asthma patients tend to use herbal medicines as one of the common therapies of complementary and alternative medicine. However, these therapies often have insufficient evidence for their effectiveness in asthma [5].

Members of the public obtain information about their own or their children’s conditions or diseases from different sources, including health professionals (such as treating physicians, physiotherapists, nurses, health educators, pharmacists, or lab technologists), and media of different types (television, radio, newspapers, magazines, etc.), but most recently they have come to obtain this information through the Internet [6]. This study aimed to determine the knowledge, attitude, and perception of parents toward using herbal medicines for the treatment of asthma in their children in the Najran Region.

## Materials and methods

This was a cross-sectional study with the research setting as Najran region, Saudi Arabia. The study population was a purposive convenience sample that included all parents of children with asthma in the Najran region.

Data collection and instrument

The participants received an electronic self-administered survey via social media channels. The survey instrument comprised a set of questions gathered from prior studies with comparable research aims. The questionnaire sheet contained three parts.

First part

The first contained parents’ demographic characteristics such as gender, age, marital status, nationality, educational level, work, and region of residence.

Second Part

This contained questions regarding asthma including does the parent personally suffer from asthma, do your children suffer from asthma, how many of your children suffer from asthma, and questions to assess the parent’s knowledge about asthma and what their sources of information about asthma are.

Third Part

This contained questions about herbal medicines, including have you ever used them to treat one of your children’s asthma, determining the type of herbs and folk remedies, the age of the child, does he used them with medical treatments, the extent of their use, belief in their effectiveness as a treatment, the source of knowledge about herbal medicines, what are the reasons for the tendency to use them, and whether complications or side effects occur after using them.

Ethical considerations

All ethics issued prior to conducting the study have been considered, and the data that were collected from the questionnaire remain confidential and no personal proof in any form will be required. Consent to participate in the questionnaire was obtained before starting it.

Pilot study

The pilot study was conducted on 10% of the total samples of parents with asthmatic children to ensure the clarity of the tools and confirm that the necessary changes were made. It served as an estimate for the approximate time needed to find any problems with data collection. After obtaining the results of the pilot study, modification of tools will be done. Those parents were excluded from the actual study to prevent contamination of the result.

Data management (analysis)

Data were analyzed with IBM SPSS Statistics for Windows, Version 22 (Released 2013; IBM Corp., Armonk, New York, United States). The sample and outcome variables were summarized using the descriptive statistics of frequencies, percentages, means, and standard deviations. Data were analyzed by type of provider as appropriate, and the Pearson chi-square test was used to compare the observed data, and the paired sample T-test was used to test the difference between the means of the pretest and post-test.

## Results

Table 1 showed that in terms of age, the majority of participants fall within the 18-25 age group, accounting for 34.1% of the total. This is followed by the 26-35 age group at 28.0%, the 36-45 age group at 24.3%, and those over 45 at 13.6%. Gender-wise, there is a fairly even split between male and female participants, with males making up 45.3% and females 54.7% of the total. The vast majority of participants (99.1%) are of Saudi nationality, with only a small percentage (0.9%) being non-Saudi. In terms of location, the majority of participants are from Najran, accounting for 90.7% of the total. The remaining participants are spread across various other locations, with no single location dominating the distribution. With regard to the education level, the majority of participants have attained a university degree, making up 67.3% of the total. This is followed by those with a high school education at 21.0%, and those with a master's or Ph.D. at 5.1% and 1.4% respectively. When it comes to occupation, civilians make up the largest percentage at 43.9%, followed by students at 26.2% and those in free business at 8.4%. Lastly, in terms of marital status, the majority of participants are married, accounting for 83.6% of the total. This is followed by those who are widowed at 9.3% and divorced at 7.0%.

**Table 1:** Sociodemographic characteristics of participants (n=214).

Parameter		No.	%
Age	18-25	73	34.1
	26-35	60	28.0
	36-45	52	24.3
	More than 45	29	13.6
Gender	Male	97	45.3
	Female	117	54.7
Nationality	Saudi	212	99.1
	Non-Saudi	2	.9
Location	Badr Al-Janoub	2	.9
	Habona	1	.5
	Khabash	3	1.4
	Sharurah	12	5.6
	Najran	194	90.7
	Yadmah	2	.9
Education Level	primary	5	2.3
	middle	6	2.8
	High school	45	21.0
	University	144	67.3
	Master's	11	5.1
	Ph.D	3	1.4
Occupation	free businees	18	8.4
	military	12	5.6
	Incitant	27	12.6
	Civilian	94	43.9
	retired	7	3.3
	student	56	26.2
Marital Status	Married	179	83.6
	Divorced	15	7.0
	Widowed	20	9.3

According to the data provided in Table 2, 15.0% of the respondents personally suffer from asthma, while 85.0% do not. Additionally, 25.7% of the respondents indicated that one of their children suffers from asthma, while 74.3% stated that their children do not suffer from asthma. For those who answered "yes" to the question about their children suffering from asthma, the data are further broken down. 15.9% specified that one child suffers from asthma, 5.1% specified two children, 1.4% specified three children, 1.9% specified four children, and 1.4% specified five children.

**Table 2:** Participants characteristics regarding asthma (n=214).

Parameter		No.	%
Do you personally suffer from asthma?	Yes	32	15.0
	no	182	85.0
Does one of your children suffer from asthma (chest allergies):	Yes	55	25.7
	no	159	74.3
If your previous answer is (yes), specify the number	1.0	34	15.9
	2.0	11	5.1
	3.0	3	1.4
	4.0	4	1.9
	5.0	3	1.4

Table 3 revealed that the statement "Asthma is a chronic lung disease whose severity varies from one person to another and there is a treatment for it" received a 43.0% "Strongly Agree" response, 41.1% "Agree," 13.6% "Neutral," 1.9% "Disagree," and 0.5% "Strongly Disagree." Similarly, the statement "Asthma is caused by a combination of genetic and environmental factors" received a 45.8% "Strongly Agree" response, 38.8% "Agree," 13.1% "Neutral," 1.4% "Disagree," and 0.9% "Strongly Disagree." The data also show that the statement "Asthma symptoms are dry cough, shortness of breath, and wheezing" received a 51.4% "Strongly Agree" response, 37.9% "Agree," 9.3% "Neutral," 0.9% "Disagree," and 0.5% "Strongly Disagree." However, there are also areas of contention within the data. For example, the statement "Using asthma medications does not cause addiction to them" received a 36.0% "Strongly Agree" response, 32.7% "Agree," 22.0% "Neutral," 5.1% "Disagree," and 4.2% "Strongly Disagree."

**Table 3:** Knowledge of participants of asthma (n=214).

Parameter	Strongly Agree	Agree	Neutral	Disagree	Strongly Disagree
Asthma is a chronic lung disease whose severity varies from one person to another and there is a treatment for it	92 43.0%	88 41.1%	29 13.6%	4 1.9%	1 .5%
Asthma is caused by a combination of genetic and environmental factors	98 45.8%	83 38.8%	28 13.1%	3 1.4%	2 .9%
Asthma symptoms are dry cough, shortness of breath, and wheezing	110 51.4%	81 37.9%	20 9.3%	2 .9%	1 .5%
Asthma symptoms often become more severe during the night and early morning	96 44.9%	71 33.2%	37 17.3%	6 2.8%	4 1.9%
Asthma attacks may be serious and life-threatening	106 49.5%	66 30.8%	35 16.4%	6 2.8%	1 .4%
Asthma medications are very important for controlling symptoms and preventing asthma attacks	110 51.4%	72 33.6%	26 12.1%	4 1.9%	2 .9%
Asthma medications include bronchodilators and cortisone	92 43.0%	77 36.0%	33 15.4%	8 3.7%	4 1.9%
Using asthma medications does not cause addiction to them	77 36.0%	70 32.7%	47 22.0%	11 5.1%	9 4.2%

As shown in Table 4, the majority of respondents (51.4%) reported obtaining information about asthma from doctors. Family and friends were also a significant source of information, with 49.5% of respondents citing them as a source. Social media and internet sites were also mentioned by a substantial number of respondents, at 38.8% and 28.5% respectively. Awareness brochures were the least common source of information, cited by 24.3% of respondents. Use of Herbs and Folk Medicines for Asthma Treatment: A notable portion of respondents (35.5%) reported using herbs or folk medicines to treat asthma in their children. Among those who used herbal treatments, a variety of specific remedies were mentioned, such as male gum, guava leaf, anise, sesame oil, black seed, and others. The most common treatment is FX, with 40 individuals (52.6%) using it. Following closely behind are sesame oil and anise, with 39 (51.3%) and 37 (48.7%) individuals using them, respectively. Guava leaf, male gum, and other treatments also have a significant number of individuals using them, ranging from 30 (39.5%) to 36 (47.4%). The age distribution of children who used medicinal herbs varied, with a significant proportion falling in the 5-12 years age group (38.2%). Regarding attitudes and beliefs about herbal treatments, a substantial portion of respondents (44.9%) reported not using medicinal herbs at all. Despite this, 41.6% of respondents expressed belief in the idea of taking herbs as a treatment, while 17.8% disagreed with this notion. A minority of respondents (13.1%) reported experiencing complications or side effects after using medicinal herbs.

**Table 4:** Attitude of participants of use of herbal medicine in the treatment of asthma (n=214).

Parameter		No.	%
What is your source of information about asthma?	Family and friends	106	49.5
	the doctor	110	51.4
	Social media	83	38.8
	Internet sites	61	28.5
	Awareness brochures	52	24.3
Have you ever used herbs or folk medicines to treat asthma in one of your children?	Yes	76	35.5
	no	138	64.5
If your answer is yes to the previous question, specify the type of treatment used?	Male gum	36	47.4
	Guava leaf	30	39.5
	Anise	37	48.7
	Sesame oil	39	51.3
	Black seed	27	35.5
	Indian installment	11	14.5
	Cress love	14	18.4
	FX	40	52.6
	Other	36	47.4
How old is the child who used medicinal herbs?	0-12 months	6	7.9
	1 - 4 years	21	27.6
	5-12 years	29	38.2
	13-18 years	20	26.3
Do you use medicinal herbs with medications prescribed by a doctor?	Sometimes alone and sometimes in conjunction with medical medications	42	19.6
	Use it alone	37	17.3
	Yes, I use it in conjunction with medical medications	39	18.2
	I don't use it	96	44.9
To what extent do you use herbs and folk medicines to treat asthma?	always	21	9.8
	sometimes	76	35.5
	Scarcely	37	17.3
	I don't use it at all	80	37.4
Do you believe in the idea of taking herbs as a treatment?	Yes	89	41.6
	neutral	87	40.7
	no	38	17.8
How did you know about folk and herbal treatment?	Relatives and friends	126	58.9
	Internet sites	50	23.4
	Popular processor	24	11.2
	Social media	77	36.0
What are the reasons for you turning to herbs and folk medicines rather than turning to medical medications?	Medical treatment is ineffective	43	20.1
	Society and friends encourage the use of medicinal herbs	74	34.6
	Treatment is available, but it is not known how to use it	14	6.5
	I trust folk medicine more than modern medicine	9	4.2
	Medical treatment is expensive	13	6.1
	Medical treatment is not available	21	9.8
	Lack of a specialist doctor	33	15.4
	Medicinal herbs are less harmful and safer than medical treatment	62	29.0
Did any complications or side effects occur after using medicinal herbs?	Yes	28	13.1
	no	186	86.9

Based on the data provided in Table 5, it can be observed that the age group 18-25 has the highest percentage of good scores (26.6%), while the age group 45 and more has the lowest percentage of good scores (8.4%), with significant association (p-value=0.012). In terms of marital status, married individuals have the highest percentage of good scores (52.8%), followed by widows (6.5%) and divorced individuals (5.1%), with no significant association. In terms of gender, females have a slightly higher percentage of good scores (32.7%) compared to males (31.8%), with no significant association. Among the different nationalities, Saudis have a higher percentage of good scores (63.6%) compared to non-Saudis (0.9%), with no significant association. In terms of location, Najran has the highest percentage of good scores (59.3%), followed by Sharoura (3.7%) and Badr Al-Janoub (0.5%), with no significant association. In terms of education level, individuals with a university degree have the highest percentage of good scores (47.2%), followed by high school (10.7%) and master's degree (3.3%). In terms of occupation, civilians have the highest percentage of good scores (26.2%), followed by students (22.0%), with significant association (p-value=0.006)

**Table 5:** Association between sociodemographic characteristics and knowledge of participants of use of herbal medicine in the treatment of asthma (n=214).

Parameter		Knowledge score		Total (N=214)	p-value
		Poor	good		
Age	18-25	16	57	73	0.012
		7.5%	26.6%	34.1%	
	26-35	23	37	60	
		10.7%	17.3%	28.0%	
	36-45	26	26	52	
		12.1%	12.1%	24.3%	
	45 and more	11	18	29	
		5.1%	8.4%	13.6%	
marital status	Married	66	113	179	0.631
		30.8%	52.8%	83.6%	
	Divorced	4	11	15	
		1.9%	5.1%	7.0%	
	widow	6	14	20	
		2.8%	6.5%	9.3%	
Gender	Male	29	68	97	0.118
		13.6%	31.8%	45.3%	
	Female	47	70	117	
		22.0%	32.7%	54.7%	
Nationality	Saudi	76	136	212	0.292
		35.5%	63.6%	99.1%	
	Non-Saudi	0	2	2	
		0.0%	0.9%	0.9%	
Location	Badr Al-Janoub	1	1	2	0.614
		0.5%	0.5%	0.9%	
	Habona	1	0	1	
		0.5%	0.0%	0.5%	
	Khabash	2	1	3	
		0.9%	0.5%	1.4%	
	Sharoura	4	8	12	
		1.9%	3.7%	5.6%	
	Najran	67	127	194	
		31.3%	59.3%	90.7%	
	Yadmah	1	1	2	
		0.5%	0.5%	0.9%	
Education Level	Primary	3	2	5	0.193
		1.4%	0.9%	2.3%	
	Middle	3	3	6	
		1.4%	1.4%	2.8%	
	High school	22	23	45	
		10.3%	10.7%	21.0%	
	University	43	101	144	
		20.1%	47.2%	67.3%	
	Master's	4	7	11	
		1.9%	3.3%	5.1%	
	PHD	1	2	3	
		0.5%	0.9%	1.4%	
Occupation	Military	4	8	12	0.006
		1.9%	3.7%	5.6%	
	Incitant	13	14	27	
		6.1%	6.5%	12.6%	
	Free business	7	11	18	
		3.3%	5.1%	8.4%	
	Civilian	38	56	94	
		17.8%	26.2%	43.9%	
	Retired	5	2	7	
		2.3%	0.9%	3.3%	
	Student	9	47	56	
		4.2%	22.0%	26.2%	

## Discussion

The survey data on the knowledge, attitude, and perception of parents in the Najran region towards the use of herbal medicine in the treatment of asthma in their children provides valuable insights into the prevalent practices and beliefs within the community. This discussion aims to analyze the key findings and implications of the survey and highlight potential areas for further research and intervention.

Knowledge of herbal medicine for asthma treatment

The data indicate that a significant proportion of parents have knowledge about herbal medicine for treating asthma in their children. This is evident from the reported use of various herbal remedies such as FX, black seed, sesame oil, guava leaves, and others. More than one-third of the participants sometimes use herbal medicine with their asthmatic children, and nearly 10% always use it. This is consistent with studies that showed participants relying on the use of traditional medicine [7,8]. Similarly, studies have shown that a considerable prevalence of complementary and alternative medicine (CAM) use has been seen in the context of asthma, with reported rates ranging from 6% to 84% [9]. Nevertheless, there is a scarcity of studies that have assessed parental knowledge, attitudes, and behaviors about self-medication for their children [10,11].

Knowledge of asthma was good in our study, as the rate of correct responses regarding asthma ranged between 70% and 90% among the participants, which is inconsistent with research conducted in Saudi Arabia in 2018 that revealed a significant lack of awareness among 55.3% of parents [12].

Attitudes and perception toward herbal medicine

The survey reveals a moderate attitude and perception toward herbal medicine for asthma treatment. While a notable percentage of parents reported using herbal treatments for their children's asthma, a substantial portion also expressed skepticism or lack of belief in the efficacy of herbal remedies. The reasons for turning to herbal medicine, such as societal encouragement, perceived safety, and cost, highlight the complex interplay of cultural, economic, and healthcare system factors influencing parental attitudes [13].

Throughout history, medicinal plants have been used in many civilizations as a primary resource for medicinal purposes. Research suggests that around 80-85% of individuals living in both developed and developing nations depend on traditional medicine as their primary source of healthcare. It is widely believed that a significant portion of traditional treatment includes the use of plant extracts or their active components [14]. Especially in asthma, for instance, the potential of sesame oil to mitigate pulmonary edema and bronchial neutrophilic inflammation in cases of allergic asthma is suggested via its ability to reduce systemic IgE levels [15]. According to research, it was shown that the ethanol extract of N. sativa had a significant ability to suppress the release of histamine from mast cells in peritoneal Wistar rats. This inhibitory action was seen to be directly related to the concentration of the extract. Nigella sativa demonstrates efficacy as an anti-inflammatory agent on mast cells via the suppression of histamine production, while exhibiting no harmful effects on mast cells. Nigella sativa may be regarded as a promising intervention for the treatment and prevention of asthma [16].

The data indicating that a minority of parents reported experiencing complications or side effects after using medicinal herbs underscore the need for increased awareness and education about the potential risks associated with herbal treatments. Addressing these concerns is crucial to ensuring the safety and well-being of children with asthma who are exposed to herbal remedies [17].

The findings have several implications for healthcare practices and policies in the Najran region. Firstly, there is a need for healthcare providers to engage with parents and the community to better understand their knowledge, attitudes, and practices regarding herbal medicine for asthma treatment. This can facilitate the development of culturally sensitive and effective interventions that integrate traditional and modern medical approaches. Furthermore, healthcare policies and programs should prioritize the dissemination of evidence-based information on the safe and appropriate use of herbal medicine for asthma. This can involve promoting dialogue between healthcare professionals, traditional healers, and parents, as well as enhancing public awareness campaigns about the potential benefits and risks of herbal treatments [18].

The primary limitation of this research is that to date there is no similar study done in the Middle East, even worldwide there are scarce studies on this topic, which made it hard for us to compare our results. Also, this study had a relatively limited sample size (n=214), which only covers a certain community in Najran. Therefore, the results of this research cannot be extrapolated to include the whole population of individuals with allergies.

## Conclusions

In conclusion, the survey data on the knowledge, attitude, and perception of parents in the Najran region toward the use of herbal medicine in the treatment of asthma in their children offer valuable insights that can inform targeted interventions, healthcare policies, and future research. By addressing the complexities of traditional and modern healthcare practices, there is an opportunity to improve the overall management and outcomes of asthma in children within the community. However, there are limitations which should be considered.
